# Role of IL-2, IL-6, and TNF-α as Potential Biomarkers in Ischemic Heart Disease: A Comparative Study of Patients with CAD and Non-CAD

**DOI:** 10.3390/medsci13020040

**Published:** 2025-04-04

**Authors:** Ahmed E. Altyar, Shilpa Bhardwaj, Nehmat Ghaboura, Priya Kaushik, Sattam Khulaif Alenezi, Mohammed Jaffar Sadiq Mantargi, Muhammad Afzal

**Affiliations:** 1 Department of Pharmacy Practice, Faculty of Pharmacy, King Abdulaziz University, P.O. Box 80260, Jeddah 21589, Saudi Arabia; aealtyar@kau.edu.sa; 2 Pharmacy Program, Department of Pharmacy Practice, Batterjee Medical College, P.O. Box 6231, Jeddah 21442, Saudi Arabia; 3 Department of Neurochemistry, Institute of Human Behaviour and Allied Sciences Hospital, Delhi 110095, India; 4 Pacific Institute of Medical Sciences, Udaipur 313015, India; 5 Department of Pharmacology and Toxicology, College of Pharmacy, Qassim University, Buraydah 51452, Saudi Arabia; 6 Pharmacy Program, Department of Pharmaceutical Sciences, Batterjee Medical College, P.O. Box 6231, Jeddah 21442, Saudi Arabia

**Keywords:** cytokines, coronary artery disease, ischemic heart disease, inflammation

## Abstract

**Background:** Ischemic heart disease (CAD), a leading global health burden, arises primarily from atherosclerosis, an inflammatory condition characterized by lipid accumulation and metabolic dysregulation. The precise contribution of inflammatory cytokines (IL-2, IL-6, and TNF-α) to CAD pathogenesis remains an area of significant research. **Aim:** The primary aim of this study is to examine the IL-2, IL-6, and TNF-α in patients with coronary artery disease (CAD) and compare them with Non-CAD individuals to evaluate their potential as diagnostic biomarkers for CAD. **Methodology:** A prospective observational study was conducted over 3 years, involving 100 participants divided into CAD and non-CAD groups. Blood samples were isolated and analyzed for IL-2, IL-6, and TNF-α levels utilizing ELISA kits. Biochemical parameters, including lipid profiles, were also assessed. **Results:** This study observed significantly elevated IL-6 in patients with CAD compared with controls, while IL-2 and TNF-α levels did not reach statistical significance. The CAD group exhibited dyslipidemia characterized by elevated triglycerides and reduced HDL. Furthermore, the CAD group demonstrated alterations in biochemical parameters, including lower albumin and calcium levels, higher urea and uric acid levels, and an elevated erythrocyte sedimentation rate. These findings suggest a systemic inflammatory state and metabolic disturbances in patients with CAD. **Conclusions:** This study highlights IL-6 as a potential biomarker and key player in CAD pathogenesis. These findings warrant further investigation into the therapeutic potential of targeting inflammatory pathways for cardiovascular risk reduction.

## 1. Introduction

Cardiovascular diseases (CVDs) constitute a significant global health burden, accounting for approximately one-third of all global mortality. Within the spectrum of CVDs, ischemic heart disease (e.g., CAD) emerges as the most prevalent condition. Coronary artery disease (CAD) poses a substantial threat to sustainable progression in the 21st century. Furthermore, a growing population of individuals surviving non-fatal CAD experiences chronic disabilities and a diminished quality of life. The primary etiological factor underlying CAD is atherosclerosis [1]. Atherosclerosis, the primary cause of cardiovascular disease, is an inflammatory condition of the arteries characterized by lipid accumulation and metabolic dysregulation. Multiple risk factors, including smoking, hypertension, and diabetes, contribute significantly to its development [1,2]. Notably, a substantial portion of the at-risk population exhibits multiple risk factors, while a minority remains free from them. This complex, immune-mediated process involves lipid infiltration into the arterial intima, leading to the development of atherosclerotic plaques. Consequent narrowing of the arterial lumen and endothelial dysfunction disrupt myocardial blood flow, creating an oxygen supply-demand mismatch. Plaque rupture can trigger acute coronary thrombosis, resulting in severe arterial occlusion, abrupt hypoperfusion, and, ultimately, myocardial infarction [1,3]. Atherosclerosis, characterized by chronic inflammation, exhibits a complex interplay of inflammatory cells, cytokines, and chemokines throughout its progression. This inflammatory milieu contributes significantly to the development and complications of the disease [4]. Furthermore, accumulating evidence suggests a crucial role for cytokines and other endogenous mediators in the pathogenesis of various myocardial dysfunctions, highlighting their pivotal involvement in the development of heart disease [5]. The development of CVD, encompassing CAD, is compared with marked elevation of proinflammatory cytokines, such as tumor necrosis factor-alpha (TNF-α), interferon-gamma (IFN-γ), interleukin-1 beta (IL-1β), interleukin-2 (IL-2), and interleukin-6 (IL-6). These cytokines serve a crucial role in atherogenesis, characterized by the formation of atherosclerotic plaques within the tunica intima of arterial walls. This process initiates with the accumulation of lipids and immune cells, such as macrophages, mast cells, and T cells, forming a fatty streak [6,7]. Activated macrophages within the atheroma release a cascade of inflammatory mediators, including cytokines and chemokines, leading to endothelial dysfunction and tissue damage. Furthermore, these cytokines promote the expression of pro-atherogenic genes, facilitating the retention and infiltration of lipids within the vessel wall. The oxidation or enzymatic modification of low-density lipoprotein (LDL) within the intima stimulates the release of phospholipids, activating endothelial cells and amplifying the inflammatory response. This results in the recruitment and differentiation of monocytes into macrophages, which further exacerbate inflammation by secreting cytokines and growth factors, thus constituting a crucial for atherosclerotic lesions [6].

Inflammation, mediated by cytokines and chemokines, serves a crucial role in the pathogenesis of myocardial dysfunction and subsequent adverse cardiac remodeling, as demonstrated by extensive experimental and clinical evidence. Circulating levels of pro-inflammatory cytokines are consistently observed to be significantly elevated in heart failure patients, irrespective of their specific heart failure phenotype, whether it be heart failure. Furthermore, a notable exacerbation of cytokine levels is evident in patients experiencing acute decompensation events, and these elevated levels have been shown to possess prognostic significance in predicting clinical outcomes [8].

Study evaluating inflammatory biomarkers (e.g., IL-6, TNF-α) for CAD risk prediction have yielded inconsistent results. While some demonstrate associations between these biomarkers and increased CAD risk, their incremental value in predictive models is debated [9]. This investigation aims to examine the potential of emerging biomarkers, specifically IL-2, IL-6, and TNF-α, as diagnostic indicators for CAD within our population. Given the escalating prevalence of CAD, early identification of at-risk individuals through biomarker analysis would facilitate the timely implementation of preventative strategies and potentially improve clinical outcomes.

## 2. Methodology

### 2.1. Study Design

A prospective, observational study was conducted over a three-year period (May 2019–April 2022) at Tertiary Care Hospital, involving collaborative efforts between the Departments of Biochemistry and the Faculty of Medicine and Health Sciences. One hundred patients were recruited for this investigation with informed consent. The investigation was conducted following ethical principles and adhered to rigorous ethical and regulatory guidelines, including the ICH-GCP, International Organization for Standardization (ISO) 14155:2020, and the Declaration of Helsinki. Patients were categorized into two groups: a study group comprising 50 patients aged 35 years and above with a confirmed diagnosis of stable CAD and a control group consisting of 50 age- and sex-matched individuals with no clinical or electrocardiographic evidence of CAD.

### 2.2. Inclusion Criteria

Subjects with a stable diagnosis of CAD, as defined by the WHO, were recruited for this investigation. Inclusion criteria included a stable clinical course with no recent evidence of acute coronary events, as determined by serial electrocardiograms (ECGs), cardiac enzyme levels (Troponin T, CK-MB), and other relevant investigations (e.g., treadmill stress test (TmT), echocardiography (ECHO), thallium scintigraphy, coronary angiography) as clinically indicated. To maintain participant safety and ethical considerations, prescribed medications (including lipid-lowering agents, antihypertensives, and antidiabetics) were not discontinued during the study.

### 2.3. Exclusion Criteria

Exclusion criteria encompass individuals with a recent history of acute myocardial infarction (MI), stroke, or transient ischemic attack within the preceding eight weeks. Patients experiencing febrile episodes, including those undergoing tuberculosis treatment, are also excluded. Furthermore, individuals with ongoing chronic inflammatory conditions such as rheumatoid arthritis, systemic lupus erythematosus, pelvic inflammatory disease, inflammatory bowel disease, or connective tissue disorders are ineligible for participation. This exclusion extends to individuals with chronic lung diseases, including chronic obstructive pulmonary disease, chronic liver diseases such as cirrhosis, and chronic kidney disease. Additionally, individuals suffering from debilitating illnesses, such as cancer, and those who have undergone coronary angioplasty or surgery within the past six months were not considered for the study.

This study included 100 participants, consisting of 64 males and 36 females, divided into two groups, i.e., study and control. The demographic analysis (Table 1) of the study population reveals that the average age of participants in the CAD group was 54.4 years with a standard deviation (SD) of 11.5, while the average age in the Non-CAD group was 51.0 years with an SD of 10.8. The *p*-value for the age difference between the two groups was 0.1339, indicating that the difference is not statistically significant. Regarding gender distribution, 66.0% of patients in the CAD group were male and 34.0% were female, whereas in the Non-CAD group, 62.0% were male and 38.0% were female. The *p*-value for the gender distribution between the two groups is 0.6769, suggesting that the difference is not statistically significant. The analysis shows no statistically significant differences in age and gender distribution between the CAD and Non-CAD groups.

Regarding alcohol consumption, out of the total 50 participants, 20% were alcohol consumers (n = 10), while the remaining 80% (n = 40) were non-consumers. Regarding smoking status, 42% of the participants (n = 21) were smokers, whereas 58% (n = 29) were non-smokers. In terms of hypertension status, 32% of the participants were hypertensive, while 68% were non-hypertensive. Similarly, when assessing diabetes mellitus status, 16% of the participants (n = 8) had diabetes, while the majority, 84% (n = 42), were non-diabetic. These findings provide a comprehensive overview of the distribution of various parameters in the CAD group.

### 2.4. Sample Collection and Storage

Following an overnight fast, venous blood specimens were obtained from the antecubital vein of all patients under sterile conditions. Plasma samples were preserved at a temperature of −80 °C until assayed. The biochemical analysis was performed using standard commercially available diagnostic kits. A blood sample was collected into the following separate vials: plain vials for routine biochemical analyses including a lipid profile and EDTA-containing vials for the quantification of IL-6, IL-2, TNF-α, NO, and endothelin. Specifically, routine biochemical analyses, including total cholesterol, triglycerides, and high-density lipoprotein (HDL) cholesterol, blood glucose levels, albumin levels, renal function markers, liver function markers, determined using established laboratory techniques on a fully automated spectrophotometric analyzer (AU480, Beckman Coulter Pvt Ltd., Singahalli, India), with commercially available reagent kits. LDL cholesterol was calculated using the Friedewald equation. The concentration of TNF-α in plasma was quantified using a human TNF-α ELISA kit. A microplate reader (Merilyzer Eiaquant, Meril Life Sciences Pvt. Ltd., Vapi, India) was used to determine the plasma concentration of the tested proteins from appropriately calibrated recombinant human proteins. Calibration was performed with reactivity towards natural and recombinant forms of human TNF-α. Similarly, plasma levels of Endothelin, IL-2, and IL-6 were assessed using respective human ELISA kits, each specifically designed to detect corresponding cytokines. Nitric oxide (NO) levels were estimated using a modified Griess reaction [10].

### 2.5. Statistical Analysis

Data analysis commenced with descriptive statistics to summarize the key features of the dataset. To assess treatment effects, independent sample t-tests were employed to compare means between groups, assuming normally distributed data. The non-parametric Chi-square test was utilized to compare the distributions of the two groups. Correlation analysis evaluated the strength and direction of relationships between continuous variables using the Pearson correlation coefficient with a *p*-value. These statistical methods are widely recognized and frequently applied within the context of clinical trials. Their primary applications encompass evaluating treatment efficacy and safety, examining the associations between patient characteristics and clinical outcomes, and identifying potential predictors of treatment response.

## 3. Results

### 3.1. Inflammatory Markers

**Figure 1:**
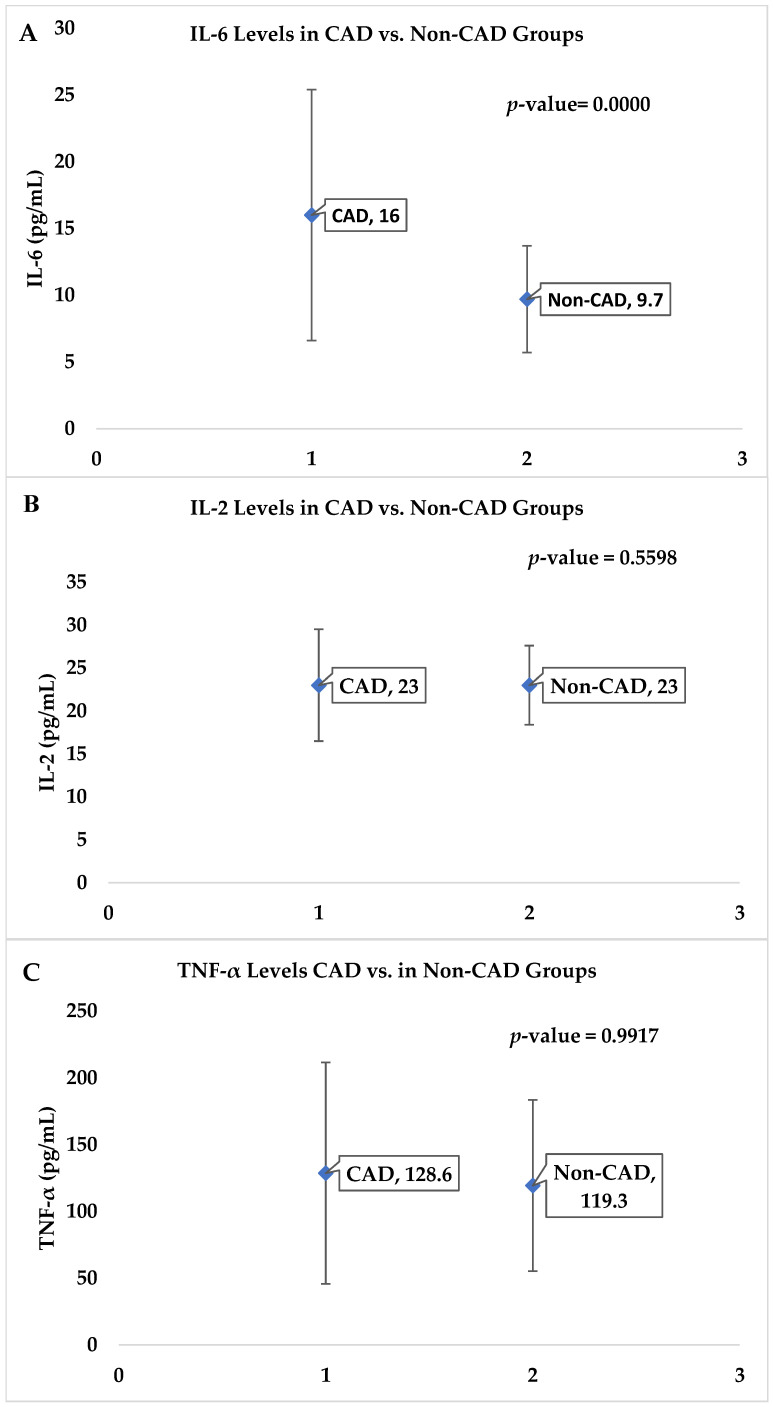
(**A**–**C**) Comparison of the CAD and Non-CAD groups for inflammatory markers, (**A**) IL-6, (**B**) IL-2, and (**C**) TNF-α.

The descriptive analysis and comparison of the CAD and Non-CAD groups revealed several significant findings. The parameter NO showed a significant difference between the groups, with the CAD group having an average value of 16.4 (SD = 21.1) and the Non-CAD group having an average value of 17.7 (SD = 13.8) with a *p*-value of 0.0137. Inflammatory markers such as IL-6 were markedly elevated in the CAD group (16.0 ± 9.4) compared with the Non-CAD group (9.7 ± 4.0), with a *p*-value of 0.0000 (Figure 1A). No statistically significant variations in IL-2 and TNF-α were observed between the experimental groups, as depicted in Figure 1B,C. The CAD group had higher levels of IL-2 (23.0 ± 6.5) and TNF-α (128.6 ± 82.9) compared with the Non-CAD group; no statistically significant differences were observed between the groups, as indicated by *p*-values of 0.5598 and 0.9917, respectively.

Nitric oxide (NO) levels were slightly lower in patients with CAD (16.4 ± 21.1) than in Non-CAD individuals (17.7 ± 13.8), with a *p*-value of 0.0137.

### 3.2. Lipid Profile

Lipid parameters also showed notable differences. Triglycerides (TG) were significantly higher in the CAD group (169.0 ± 88.5) than in the Non-CAD group (124.1 ± 34.9), with a *p*-value of 0.0012. HDL levels were markedly reduced in the CAD group (27.7 ± 6.8) compared to the Non-CAD group (41.2 ± 5.5), with a *p*-value of 0.0000. Cholesterol (chol200) levels were lowered in the CAD group (147.1 ± 44.9) compared to the Non-CAD group (162.3 ± 35.8), with a *p*-value of 0.0531, which is close to being statistically significant. No statistically significant intergroup variation was observed in LDL levels between the groups (*p* = 0.3075).

### 3.3. Biochemical Markers

Blood glucose levels were significantly higher in the CAD group (113.5 ± 44.9) as compared to the Non-CAD group (94.0 ± 13.5), with a *p*-value = 0.0949. Significant alterations in metabolic parameters were observed in the CAD group, including lower albumin levels (3.7 ± 0.4) compared to the Non-CAD group (3.9 ± 0.4), with a *p*-value = 0.0103. Calcium (Cal) levels were also significantly reduced in patients with CAD (9.7 ± 0.6) compared to the Non-CAD group (10.0 ± 0.6), with a *p*-value = 0.0283.

Renal function markers, including urea and uric acid, were elevated in patients with CAD. Urea levels were significantly higher in CAD individuals (32.2 ± 25.8) than in the Non-CAD group (24.7 ± 9.7), with a *p*-value = 0.0132. Uric acid levels were also increased in the CAD group (5.0 ± 1.5) compared to the Non-CAD group (4.2 ± 1.1), with a *p*-value = 0.0092.

### 3.4. Hematological Markers

Erythrocyte sedimentation rate (ESR), an indicator of systemic inflammation, was significantly elevated in patients with CAD (32.7 ± 17.7) compared to the Non-CAD group (18.7 ± 10.8), with a *p*-value = 0.0000. Total leukocyte count (TLC) was also significantly higher in patients with CAD (6550.0 ± 1950.4) than in the Non-CAD group (5750.0 ± 1242.0), with a *p*-value = 0.0162. However, hemoglobin (Hb) levels were comparable between the groups (*p* = 0.7193).

### 3.5. Liver Function Markers

Liver enzymes such as ALT, AST, and ALP did not show significant differences between the groups. The CAD group had ALT levels of 38.0 ± 26.4 compared to the Non-CAD group (35.1 ± 17.7), with a *p*-value = 0.7353. AST levels were 38.7 ± 16.2 in the CAD group compared to 35.5 ± 13.4 in the Non-CAD group, with a *p*-value = 0.3134. ALP levels were 83.0 ± 40.2 in the CAD group compared to the Non-CAD group, 87.7 ± 32.3, with a *p*-value = 0.5578. These findings suggest that there are notable biochemical differences between individuals with CAD and those without, which could have implications for understanding the pathophysiology and potential biomarkers of CAD.

### 3.6. Electrolytes and Other Parameters

Sodium (Na) levels were significantly lower in patients with CAD (140.3 ± 5.6) compared to the Non-CAD group (144.4 ± 5.4), with a *p*-value = 0.0006. Phosphorus (PHOS) levels were significantly higher in patients with CAD (3.3 ± 0.9 mg/dL) compared to the Non-CAD group (2.9 ± 0.8), with a *p*-value = 0.0067. The comparison of CAD and Non-CAD groups is shown in Table 2.

### 3.7. The iNOS Analysis

The iNOS analysis (Table 3) compared the distribution of iNOS single nucleotide polymorphism (SNP) C150T between the CAD and Non-CAD groups. In the CAD group, 70% of participants (35 out of 50) and in the Non-CAD group, 76% of participants (38 out of 50) had CC genotype/wild type. The *p*-value for this comparison is 0.4992, demonstrating that the difference in genotype distribution between the two groups is not statistically significant. Additionally, 30% of participants (15 out of 50) in the CAD group and 24% of participants (12 out of 50) in the Non-CAD group had the CT mutant genotype.

#### Assessment of CAD

ECG is the most rampant and easily available method to identify CAD. In the assessment of CAD, electrocardiogram (ECG) findings revealed that 80% of the participants (n = 40) exhibited normal ECG patterns, while only 20% (n = 10) showed abnormal findings.

Coronary angiography results demonstrated varying degrees of vessel involvement. Among the 21 participants who underwent angiography, 9.52% (n = 2) showed no significant blockage, while 38.10% (n = 8) were diagnosed with single-vessel disease. Additionally, 19.05% (n = 4) had two-vessel disease, 28.57% (n = 6) presented with three-vessel disease, and 4.76% (n = 1) were found to have four-vessel disease, indicating the severity and extent of coronary artery involvement (Table 4).

### 3.8. Correlation of Biomarkers with CAD

The correlation analysis for the CAD group shows the relationship between TNF-α, IL-6, IL2, Endo, Chol, TG, and NO, as depicted in Table 5.

In a cohort of 50 participants (n = 50), the correlation analysis between various biomarkers revealed that IL-6 and IL-2 exhibited a weak negative correlation (r = −0.045, *p* = 0.757), with a CI ranging from 0.319 to 0.236, indicating no statistical significance. Similarly, IL-6 and TNF-α showed a weak positive correlation (r = 0.092, *p* = 0.524, CI: −0.191 to 0.361), while IL-6 and Endo displayed a weak negative correlation (r = −0.139, *p* = 0.337, CI: −0.401 to 0.145), both of which were not statistically significant. IL-6 also showed weak positive correlations with Chol (r = 0.128, *p* = 0.375, CI: −0.156 to 0.393), TG (r = 0.130, *p* = 0.367, CI: −0.154 to 0.394), and HDL (r = 0.109, *p* = 0.450, CI: −0.174 to 0.376), none of which were significant. However, IL-6 and No exhibited a moderate positive correlation (r = 0.206, *p* = 0.151, CI: −0.077 to 0.458), which also lacked statistical significance.

In contrast, IL-2 and TNF-α displayed a weak positive correlation (r = 0.084, *p* = 0.564, CI: −0.199 to 0.354), while IL-2 and Endo showed a moderate negative correlation (r = −0.211, *p* = 0.142, CI: −0.462 to 0.072), both of which were not statistically significant. IL-2 also demonstrated weak negative correlations with Chol (r = −0.134, *p* = 0.353, CI: −0.398 to 0.150) and TG (r = −0.126, *p* = 0.383, CI: −0.391 to 0.158). However, IL-2 and HDL exhibited a significant moderate negative correlation (r = −0.321, *p* = 0.023, CI: −0.550 to −0.047). The correlation between IL-2 and No was nearly zero (r = −0.010, *p* = 0.945, CI: −0.288 to 0.269), indicating no association.

Furthermore, TNF-α and Endo showed a weak positive correlation (r = 0.106, *p* = 0.462, CI: −0.177 to 0.374), while TNF-α and Chol (r = 0.054, *p* = 0.708, CI: −0.228 to 0.328), TNF-α and TG (r = 0.105, *p* = 0.468, CI: −0.178 to 0.373), and TNF-α and HDL (r = 0.013, *p* = 0.930, CI: −0.266 to 0.290) all showed weak correlations with no statistical significance. TNF-α and NO exhibited a weak negative correlation (r = −0.107, *p* = 0.461, CI: −0.374 to 0.177), which was also not significant.

IL6, IL2, and TNF-α showed varying degrees of correlations with lipid parameters, NO, and Endo but were not statistically significant, except for a significant negative correlation of IL 2 with HDL.

## 4. Discussion

CAD constitutes a leading cause of mortality in developed nations, and its prevalence is escalating in developing countries. Within the Indian context, CAD accounts for a substantial 25% of all deaths, with an alarmingly early onset typically observed around the age of 45–50 years, preceding its typical presentation in Western populations by approximately a decade [11,12,13]. This premature manifestation of CAD in younger Indian individuals suggests a significant role for non-traditional risk factors, as conventional risk determinants alone cannot fully explain the observed disease burden in this population [12]. Recognizing this critical gap in our understanding, the present study explored the potential of emerging biomarkers, specifically IL-2, IL-6, and TNF-α, as valuable prognostic indicators within the Indian population. By elucidating the role of these biomarkers, we anticipate facilitating early diagnosis and the timely implementation of preventive strategies to mitigate the escalating impact of CAD in our nation.

This investigation included 100 participants (64 males and 36 females) who provided informed consent. Demographic analysis revealed no significant differences between the CAD and Non-CAD groups in age (*p* = 0.1339) and gender distribution (*p* = 0.6769). While no significant differences were observed in height (*p* = 0.1234) and weight (*p* = 0.4567), a statistically significant difference was found in BMI (*p* = 0.0118), with the CAD group exhibiting a higher BMI (26.0 ± 3.2) compared to the Non-CAD group (24.4 ± 2.3).

Emerging evidence strongly supports a pivotal role for inflammation in the progression of CVD. Inflammation, an independent predictor of CVD beyond traditional risk factors, emerges as a pivotal mechanism underlying atherogenesis. Diverse etiologies, encompassing conventional risk factors, psychological stress, autoimmune conditions, infections, and aging, converge on endothelial dysfunction, triggering a low-grade inflammatory response within the vasculature [14]. This inflammatory milieu facilitates the progression of atherosclerosis, culminating in clinical manifestations such as CAD, large artery thrombotic stroke, and cerebral aneurysms. The intricate pathogenesis of atherosclerosis, encompassing lipoprotein retention, plaque formation, and rupture, involves the orchestrated interplay of innate and adaptive immune components, including bone marrow, spleen, and circulating immune cells (leukocytes, macrophages, and lymphocytes). These elements modulate the inflammatory landscape by regulating pro- and anti-inflammatory cytokines and other protein mediators [14,15]. Atherosclerosis, a prevalent global disease, is strongly associated with pro-inflammatory cytokines. Vascular smooth muscle cells contribute significantly to the production of IL-6, a key player in atherosclerotic pathogenesis. Notably, IL-6 administration has been demonstrated to induce substantial elevations in other inflammatory cytokines, such as IL-1β and TNFα, and accelerate the formation of atherosclerotic lesions. Our study of IL6 also showed a weak positive correlation with TNF-α levels. Furthermore, elevated mRNA and protein expression of IL-6 has been consistently observed within atherosclerotic plaques and arterial walls in both human and rodent models compared to non-atherosclerotic arterial tissues [16]. IL-2 and TNF-α are key regulators of immune responses and have been implicated in atherosclerosis and CAD pathogenesis [17,18]. In our study, IL-2 levels were found to be significantly negatively correlated with HDL, which is the protective lipid.

The present study observed significantly elevated levels of the inflammatory marker IL-6 in patients with CAD (16.0 ± 9.4) compared to the Non-CAD control group (9.7 ± 4.0) (*p* < 0.0001). While higher levels of IL-2 (23.0 ± 6.5) and TNF-α (128.6 ± 82.9) were detected in the CAD group, these differences did not reach statistical significance compared to the Non-CAD group (*p* = 0.5598 and *p* = 0.9917, respectively). These outcomes are aligned with previously reported literature [17].

Proinflammatory cytokines, notably TNF-α and IL-6, exert significant influence on lipid metabolism, thereby contributing to the pathogenesis of CAD. Several well-established CAD biomarkers, such as inflammatory markers, including C-reactive protein (CRP), soluble CD40 ligand (sCD40L), myeloperoxidase (MPO), and B-type natriuretic peptide (BNP), are used in cardiovascular risk assessment. CRP is widely recognized as a marker of systemic inflammation and has strong associations with CAD progression, while sCD40L and MPO are involved in plaque instability and oxidative stress, respectively. BNP is a critical indicator of myocardial strain and heart failure risk [19,20]. Our study focused on pro-inflammatory cytokines, particularly IL-6, IL-2, and TNF-α, due to their involvement in the inflammatory cascade of atherosclerosis. The results of our research indicated that IL-6 levels are significantly higher in patients with CAD compared to controls, pointing to IL-6 as a key inflammatory mediator involved in CAD pathogenesis. Even though IL-2 and TNF-α showed an increasing trend, they did not reach statistical significance. In previous studies, IL-6 has been implicated in endothelial dysfunction, vascular inflammation, and acute coronary events, supporting its potential as a biomarker [16,17]. Our study adds to the growing body of evidence by demonstrating the association of IL-6 with CAD, highlighting the need for further research to explore its diagnostic and prognostic potential alongside conventional biomarkers.

These cytokines disrupt lipid homeostasis by inhibiting lipoprotein lipase activity, stimulating hepatic triglyceride synthesis, and impairing HDL function, leading to elevated triglycerides, decreased HDL levels, and increased LDL oxidation. In our study, we observed weak correlations of cytokines with lipid profiles, but IL2 showed a significant negative correlation with HDL levels. TNF-α and IL-6-mediated dyslipidemia, indicated by elevated TG, low HDL, and high oxidized LDL, fosters a pro-atherogenic environment by promoting endothelial dysfunction and foam cell formation, ultimately accelerating the development and progression of atherosclerotic plaques as observed in our study as well. Elevated triglycerides, reduced HDL, and increased oxidized LDL create a pro-atherogenic environment that promotes endothelial dysfunction and foam cell formation, accelerating atherosclerotic plaque progression. This mechanistic link highlights the impact of inflammation on CAD pathogenesis and reinforces the potential of anti-inflammatory and lipid-lowering strategies in reducing cardiovascular risk [21].

Our analysis revealed that lipid profiles exhibited significant differences between CAD and Non-CAD groups. Triglycerides were markedly elevated in the CAD group compared to the Non-CAD group. Conversely, HDL levels were markedly alleviated in the CAD group. According to these findings, dyslipidemia contributes to atherosclerosis and the progression of CVD. While a trend towards lower total cholesterol levels was observed in the CAD group, this difference did not attain statistical significance. No significant variations were observed in LDL cholesterol levels between the two groups. This may reflect the impact of prior medical interventions (e.g., statins). Despite this, alterations in triglyceride and HDL levels support their established roles in cardiovascular risk stratification. These findings corroborate previously published research [22].

Multiple studies have demonstrated a positive association between elevated levels of certain liver enzymes, including ALT, ALP, and gamma-glutamyltransferase (GGT), and an increased risk of CVD. Among these, GGT exhibits the strongest positive correlation with CVD despite being a non-specific marker of liver function. While ALT also shows a clear positive association with CVD, the role of ALP in CVD risk remains less defined [23]. The pathophysiology of heart failure, especially in advanced stages, often involves hepatorenal or even cardiohepatorenal syndrome, highlighting a correlation between liver dysfunction and heart failure. However, existing evidence on the relationship between abnormal liver function tests and outcomes in heart failure patients remains limited [24].

The present study observed no significant differences in ALT, AST, and ALP serum levels between patients with CAD and controls. Mean ALT levels were 38.0 ± 26.4 U/L in the CAD group and 35.1 ± 17.7 in the control group (*p* = 0.7353). Mean AST levels were 38.7 ± 16.2 U/L in the CAD group and 35.5 ± 13.4 U/L in the control group (*p* = 0.3134). Mean ALP levels were 83.0 ± 40.2 U/L in the CAD group and 87.7 ± 32.3 U/L in the control group (*p* = 0.5578). These findings do not support the hypothesis of significant hepatic dysfunction as a distinguishing feature in patients with CAD compared to controls, corroborating previous research [25].

Recent investigations have explored the association between various biochemical markers and the pathophysiology of CAD and acute ischemic stroke. Elevated levels of TLC and ESR were observed in patients with CAD relative to a control group [21]. Several studies have demonstrated that an elevated ESR is a significant predictor of CHD mortality and morbidity [20,26]. When ESR levels are elevated, circulating inflammatory mediators and fibrinogen contribute to atherogenesis and vascular dysfunction. IL-6 is a specific pro-inflammatory cytokine associated with endothelial activation and immune response modulation, whereas ESR provides a more comprehensive measure of systemic inflammation. Our study observed a significant increase in both IL-6 and ESR levels in CAD patients compared to Non-CAD individuals. ESR elevation further supports the presence of an inflammatory milieu in patients with CAD, as previous studies have linked IL-6 to CAD pathophysiology [27,28]. Furthermore, in patients experiencing myocardial infarction, significant increases in uric acid and calcium levels were reported [29].

These findings suggest that specific biochemical markers may serve as potential prognostic indicators for cardiovascular and cerebrovascular events. However, further research is imperative to elucidate the precise causal mechanisms and establish their clinical utility in risk assessment and management. Our analysis revealed significant intergroup differences in several biochemical and hematological parameters. Notably, the CAD group exhibited hypoalbuminemia, hypocalcemia, hyponatremia, hyperuricemia, and elevated urea levels compared to the Non-CAD group. Furthermore, the CAD group demonstrated leukocytosis, elevated erythrocyte sedimentation rate, increased body mass index, and hyperphosphatemia. All observed differences were statistically significant (*p* < 0.05).

iNOS exhibits multifaceted involvement in CAD. Upregulated iNOS expression within failing human cardiomyocytes suggests its potential contribution to the pathophysiology of diverse cardiac conditions, including CAD. Notably, iNOS expression has been observed in myocardial specimens from heart failure patients, predominantly within vascular endothelial and smooth muscle cells. The precise role of iNOS in myocardial ischemia-reperfusion injury remains contentious, with evidence supporting both detrimental and beneficial effects. Previously reported, iNOS, vascular inflammation, oxidative stress, and endothelial dysfunction are key factors in atherosclerosis and CAD progression. Based on this established association with inflammation, we hypothesized that iNOS expression is elevated in patients with CAD [28]. A comparative analysis was conducted to investigate the distribution of C150T SNP of iNOS between CAD and Non-CAD cohorts. The prevalence of wild-type iNOS genotype was observed in 70% (n = 35) within CAD and 76% of participants (n = 38) within Non-CAD. A statistical analysis employing a *p*-value of 0.4992 revealed no significant difference in iNOS genotype distribution between the two groups. Furthermore, a mutant allele was noted in 24% of participants (n = 12) in Non-CAD, and 30% of participants (n = 15) in CAD have CT/mutant genotype. This single-nucleotide polymorphism (SNP) has been associated with increased NO production [30]. The findings suggest that while iNOS may contribute to CAD pathogenesis in certain conditions, it may not be a distinguishing factor between patients with CAD and Non-CAD individuals in our cohort. However, larger studies would be required to establish its role. Furthermore, almost 80% of patients who underwent angio had obstruction. Other studies reported findings similar to this study, which showed normal ECG but obstruction of angio [30,31].

The correlative analysis of the investigated biomarkers revealed a spectrum of association strengths, predominantly characterized by statistically non-significant relationships. Specifically, the pro-inflammatory cytokine IL-6 demonstrated weak to moderate correlations with IL-2, TNF-α, Endo, Chol, TG, HDL, and NO. However, these associations failed to achieve statistical significance, suggesting a limited direct influence of IL-6 on these markers within the studied cohort, probably due to a smaller sample size. Notably, a weak negative correlation between IL-6 and Endo and a weak positive correlation between IL-6 and NO indicate potential trends requiring validation in larger studies. Interleukin-2 exhibited a statistically significant moderate negative correlation with HDL, indicating a possible inverse relationship whereby elevated IL-2 levels are compared with diminished HDL levels, a finding of clinical relevance due to the cardioprotective function of HDL. Correlations between IL-2 and other biomarkers, including TNF-α, Endo, Chol, TG, and NO, were observed to be weak and statistically non-significant. Similarly, TNF-α demonstrated a lack of statistically significant associations with the analyzed biomarkers. These outcomes corroborate the previously reported study [26]. Studies have shown that these cytokines are involved in inflammatory processes associated with CAD [14,15]. IL-6 has been identified as a key mediator of atherosclerosis and CVD [15]. The human immune and vascular systems have been implicated in the development of CAD, though their specific roles remain to be discovered. The present study adds to this knowledge by comparing these cytokines between patients with CAD and Non-CAD individualsin our cohort. Although IL-6 was significantly elevated, suggesting a strong association with CAD, IL-2, and TNF-α showed an increasing trend but did not reach statistical significance. It suggests that IL-6 may be a more robust biomarker for CAD in our population due to potential variability in cytokine expression. Our study contributes to a more comprehensive understanding of systemic inflammation and metabolic disturbances in CAD by analyzing these cytokines alongside lipid profiles and other biochemical parameters. Our findings reinforce IL-6 as a promising diagnostic and therapeutic target while prompting further research into the nuanced roles of IL-2 and TNF-α in cardiovascular pathology. Future research could provide deeper insights into inflammatory markers’ role in disease progression using larger sample sizes. This study’s findings regarding the association between inflammatory markers and CAD should be interpreted with caution due to several methodological limitations. The study design did not consider regional and population-specific variations in inflammatory marker levels. Moreover, the study focused on only three inflammatory markers (IL-2, IL-6, and TNF-α), omitting other key inflammatory markers involved in CAD pathogenesis, which could provide a more comprehensive perspective on the disease.

## 5. Conclusions

The investigation underscores the significant role of inflammatory markers, particularly IL-6, in the pathogenesis of CAD. Elevated levels of IL-6 were observed in patients with CAD, suggesting its potential as a diagnostic biomarker. While IL-2 and TNF-α levels exhibited a trend towards elevation in the CAD group, these differences did not show statistical significance. The findings also highlight notable biochemical differences between CAD and Non-CAD individuals, including elevated triglycerides and reduced HDL levels in the CAD group. These results emphasize the importance of inflammation and lipid metabolism in the development and progression of CAD. Further research is warranted to explore the therapeutic potential of targeting inflammatory pathways in mitigating cardiovascular risk.

## Figures and Tables

**Table 1:** Demographics characteristics.

	CAD		Non-CAD		*p*-Value (Chi-Square Test)
**Sex**	**N**	**%**	**N**	**%**	
**Male**	33	66.0%	31	62.0%	0.6769
**Female**	17	34.0%	19	38.0%	
**Grand Total**	**50**	**100.0%**	**50**	**100.0%**	
	**N**	**Average (SD)**	**N**	**Average (SD)**	***p*-value**
**Age** (years)	50	54.4 (11.5)	50	51.0 (10.8)	0.1339
**Height** (cm)	50	160.9 (5.4)	50	164.3 (6.9)	-
**Weight** (kg)	50	67.7 (11.2)	50	66.0 (9.0)	-
**BMI** (kg/m^2^)	50	26.0 (3.2)	50	24.4 (2.3)	0.0118
**Risk factors**					
**Alcohol**		**Count of Alcohol**		**Count of Alcohol2**	
Consumer (1)		10		20.00%	
Non-consumer (2)		40		80.00%	
**Grand Total**		**50**		**100.00%**	
**Smoking Status**		**Smoke**		**Count of smoke**	
Smoker (1)		21		42.00%	
Non-smoker (2)		29		58.00%	
**Grand Total**		**50**		**100.00%**	
**HTN**		**Count of HTN**		**Count of HTN2**	
Hypertensive (1)		32.00%		32.00%	
Non-hypertensive (2)		68.00%		68.00%	
**Grand Total**		**100.00%**		**100.00%**	
**DM**		**Count of DM**		**Count of DM2**	
Diabetic (1)		8		16.00%	
Non-diabetic (2)		42		84.00%	
**Grand Total**		**50**		**100.00%**	

Body mass index (BMI), Hypertension (HTN), Diabetes mellitus (DM).

**Table 2:** Descriptive analysis and comparison of the CAD and Non-CAD groups.

	CAD			Non-CAD			*p*-Value
	N	Average	SD	N	Average	SD	
**NO** (µM)	50	16.4	21.1	50	17.7	13.8	0.0137
**IL-6** (pg/mL)	50	16.0	9.4	50	9.7	4.0	0.0000
**IL-2** (pg/mL)	50	23.0	6.5	50	23.0	4.6	0.5598
**TNF-α** (pg/mL)	50	128.6	82.9	50	119.3	64.1	0.9917
**Endo** (pg/mL)	50	21.8	11.1	50	18.7	9.8	0.0787
**Chol200** (mg/dL)	50	147.1	44.9	50	162.3	35.8	0.0531
**TG150** (mg/dL)	50	169.0	88.5	50	124.1	34.9	0.0012
**HDL40** (mg/dL)	50	27.7	6.8	50	41.2	5.5	0.0000
**LDL130** (mg/dL)	50	86.5	33.5	50	93.7	33.5	0.3075
**TP** (g/dL)	50	7.3	0.7	50	7.4	0.7	0.1949
**ALB** (g/dL)	50	3.7	0.4	50	3.9	0.4	0.0103
**Cal** (mg/dL)	50	9.7	0.6	50	10.0	0.6	0.0283
**SODIUM** (mEq/L)	50	140.3	5.6	50	144.4	5.4	0.0006
**POTA** (mEq/L)	50	4.8	0.5	50	4.7	0.6	0.4419
**Urea** (mg/dL)	50	32.2	25.8	50	24.7	9.7	0.0132
**Cret** (mg/dL)	50	1.1	1.2	50	0.9	0.2	0.5747
**Uric** (mg/dL)	50	5.0	1.5	50	4.2	1.1	0.0092
**Bil** (mg/dL)	50	1.1	1.3	50	0.9	0.2	0.9143
**Gluc** (mg/dL)	46	113.5	44.9	50	94.0	13.5	0.0949
**TLC** (cells/µL)	50	6550.0	1950.4	50	5750.0	1242.0	0.0162
**ESR** (mm/h)	50	32.7	17.7	50	18.7	10.8	0.0000
**Hb** (g/dL)	50	12.6	1.7	50	12.7	1.3	0.7193
**PHOS** (mg/dL)	50	3.3	0.9	50	2.9	0.8	0.0067
**ALT** (U/L)	50	38.0	26.4	50	35.1	17.7	0.7353
**AST** (U/L)	50	38.7	16.2	50	35.5	13.4	0.3134
**ALP** (U/L)	50	83.0	40.2	50	87.7	32.3	0.5578

Nitric oxide (NO), Interleukin-6 (IL-6), Tumor necrosis factor-α (TNF-α), Endothelin (Endo), Cholesterol (Chol), Triglycerides (TG), High-density lipoprotein (HDL), Low-density lipoprotein (LDL), Total protein (TP), Albumin (ALB), Calcium (Cal), Potassium (POTA), Creatinine (Cret), Uric Acid (Uric), Bilirubin (Bil), Glucose (Gluc), Total Leukocyte Count (TLC), Erythrocyte sedimentation rate (ESR), Hemoglobin (Hb), Phosphorus (PHOS), alanine aminotransferase (ALT), aspartate aminotransferase (AST), alkaline phosphatase (ALP).

**Table 3:** iNOS analysis of CAD and Non-CAD.

	CAD		Non-CAD		*p*-Values
iNOS	N	%	N	%	
**CC**	35	70%	38	76%	0.4992
**CT**	15	30%	12	24%	

Inducible nitric oxide synthase (iNOS).

**Table 4:** Frequency distribution of CAD parameters.

	CAD	
Electrocardiogram **(ECG)**	**Count of ECG**	**% of ECG2**
Normal (1)	40	80.00%
Abnormal (3)	10	20.00%
**Grand Total**	**50**	**100.00%**
**Angio**	**Count of Angio**	**% of Angio2**
No blockage (0)	2	9.52%
Single-vessel disease (1)	8	38.10%
Two-vessel disease (2)	4	19.05%
Three-vessel disease (3)	6	28.57%
Four-vessel disease (4)	1	4.76%
**Grand Total**	**21**	**100.00%**

**Table 5:** Correlation analysis for the CAD group.

CAD Group (n = 50)	R	p-Value	C.I.
IL-6 vs. IL-2	−0.045	0.75694	0.319 to 0.236
IL-6 vs. TNF-α	0.092	0.52381	−0.191 to 0.361
IL-6 vs. Endo	−0.139	0.33739	−0.401 to 0.145
IL-6 vs. Chol	0.128	0.3746	−0.156 to 0.393
IL-6 vs. TG	0.13	0.36713	−0.154 to 0.394
IL-6 vs. HDL	0.109	0.45049	−0.174 to 0.376
IL-6 vs. NO	0.206	0.15142	−0.077 to 0.458
IL-2 vs. TNF-α	0.084	0.56412	−0.199 to 0.354
IL-2 vs. Endo	−0.211	0.14203	−0.462 to 0.072
IL-2 vs. Chol	−0.134	0.35318	−0.398 to 0.15
IL-2 vs. TG	−0.126	0.38328	−0.391 to 0.158
IL-2 vs. HDL	−0.321	0.02304	−0.55 to −0.047
IL-2 vs. NO	−0.01	0.94527	−0.288 to 0.269
TNF-α vs. Endo	0.106	0.46196	−0.177 to 0.374
TNF-α vs. Chol	0.054	0.70837	−0.228 to 0.328
TNF-α vs. TG	0.105	0.46772	−0.178 to 0.373
TNF-α vs. HDL	0.013	0.9296	−0.266 to 0.29
TNF-α vs. NO	−0.107	0.46073	−0.374 to 0.177

Nitric oxide (NO), Interleukin-6 (IL-6), Tumor necrosis factor-α (TNF-α), Endothelin (Endo), Cholesterol (Chol), Triglycerides (TG), High-density lipoprotein (HDL).

## Data Availability

The original contributions presented in this study are included in the article. Further inquiries can be directed to the corresponding author.

## Abbreviations

ALB	Albumin
ALP	Alkaline phosphatase
ALT	Alanine aminotransferase
AST	Aspartate aminotransferase
Bil	Bilirubin
BMI	Body-mass index
BNP	B-type natriuretic peptide
CAD	Coronary artery disease
Chol	Cholesterol
CRP	C-reactive protein
CVDs	Cardiovascular diseases
DM	Diabetes mellitus
ECGs	Electrocardiograms
ECHO	Echocardiography
ELISA	Enzyme-linked immunosorbent assay
Endo	Endothelin
ESR	Erythrocyte sedimentation rate
GCP	Good Clinical Practice
Hb	Hemoglobin
HDL	High-density lipoprotein
HDL	High-density lipoprotein cholesterol
HTN	Hypertension
ICH	International Council for Harmonisation of Technical Requirements for Pharmaceuticals for Human Use
IFN-γ	Interferon-gamma
IHD	Ischemic heart disease
IL-1β	Interleukin-1 beta
IL-2	Interleukin-2
IL-6	Interleukin-6
iNOS	Inducible nitric oxide synthase
ISO	International Organization for Standardization
LDL	Low-density lipoprotein
LDL	Low-density lipoprotein
MI	Myocardial infarction
MPO	Myeloperoxidase
MRS	Modified Rankin Scale
NIHSS	National Institutes of Health Stroke Scale
NO	Nitric oxide
sCD40L	Soluble CD40 ligand
TG	Triglycerides
TLC	Total Leukocyte Count
TmT	treadmill stress test
TNF-α	Tumor necrosis factor-alpha
TP	Total protein
Trop	Troponin
